# The impact of retrotransposons on castor bean genomes

**DOI:** 10.3389/fpls.2024.1397215

**Published:** 2024-07-23

**Authors:** Lin Kong, Tingting Zhang, Lei Ma

**Affiliations:** College of Life Science, Shihezi University, Shihezi, Xinjiang, China

**Keywords:** castor bean (*Ricinus communis* L.), transposable elements (TEs), genomic characteristics, evolution, retrotransposons evolution

## Abstract

Castor bean (*Ricinus communis* L.) is an important oil crop. However, the influence of transposable elements (TEs) on the dynamics of castor bean evolution awaits further investigation. This study explored the role of transposable elements in the genomes of wild castor bean accessions from Ethiopia (Rc039) and Kenya (WT05) as well as in the cultivated variety (Hale). The distribution and composition of repeat sequences in these three lineages exhibited relative consistency, collectively accounting for an average of 36.7% of the genomic sequences. Most TE families displayed consistent lengths and compositions across these lineages. The dynamics of TEs significantly differed from those of genes, showing a lower correlation between the two. Additionally, the distribution of TEs on chromosomes showed an inverse trend compared to genes. Furthermore, Hale may have originated from the ancestor of Rc039. The divergent evolutionary paths of TEs compared to genes indicate the crucial role of TEs in shaping castor bean genetics and evolution, providing insights into the fields of castor bean and plant genomics research.

## Introduction

Castor bean (*Ricinus communis* L., Euphorbiaceae, 2*n* = 20) is a valuable non-edible oil crop known for its oil-rich seeds with 45%–55% oil content, primarily rich in ricinoleic acid (Kallamadi et al., 2015; Thatikunta et al., 2016). This unique oil has extensive industrial applications in lubricants, cosmetics, coatings, inks, plastics, and biodiesel, leading to its widespread cultivation. Castor bean originates from four diversity centers based on morphological variations: (i) East Africa (Kenya and Ethiopia), (ii) West Asia (Iraq, Iran, Syria, Turkey, and Afghanistan) along with the Arabian Peninsula, (iii) India, and (iv) China (Anjani, 2012). Notably, the East African germplasm is believed to be the wild origin of contemporary castor bean, with Ethiopia and Kenya being proposed as two independent sources due to the geographical separation imposed by the Turkana Depression within the East African Rift System (Xu et al., 2021).

Transposable elements (TEs) are mobile DNA sequences found within chromosomes, capable of causing various genetic alterations such as deletions, inversions, chromosomal fusions, and more complex rearrangements (Colonna Romano and Fanti, 2022). TEs constitute a substantial portion of the genome and account for a significant proportion of DNA mass in eukaryotic cells (Schnable et al., 2009; Choulet et al., 2014). TEs possess the capacity to proliferate within a genome, subjecting them to natural selection as distinct evolutionary entities. Many TE families remain highly active and mutagenic (Eickbush and Furano, 2002; Maksakova et al., 2006), particularly in response to environmental stressors, thereby increasing genetic diversity (Horváth et al., 2017; Lanciano and Mirouze, 2018). TEs also play a significant role in influencing gene expression and regulation. They can affect host gene expression by encoding regulatory sequences necessary for their own transcription, which may also regulate nearby genes (Drongitis et al., 2019). Over time, these sequences can evolve new regulatory functions, contributing to the creation of new regulatory elements through a process known as exaptation (Brosius, 2019). A notable portion of transcription factor binding sites (TFBS) in mammalian genomes, which are crucial for various biological processes such as immune response and pregnancy, is derived from TEs (Sundaram et al., 2014; Chuong et al., 2016). Furthermore, TEs contribute to the evolution of complex gene regulatory networks by providing novel regulatory elements that can integrate into existing networks, thus driving the evolution of new regulatory functions (Britten and Davidson, 1971). The epigenetic repression of TEs, necessary to control their activity, can also spread to nearby genes, affecting their expression and potentially the organism’s fitness (Rebollo et al., 2011; Choi and Lee, 2020). In essence, TEs play a multifaceted role in driving genetic diversification and providing genetic material during genome evolution, facilitating adaptation to changing conditions (Stapley et al., 2015). However, the impact of TEs on the dynamics of castor bean evolution awaits further investigations.

In this study, we investigated the genomic characteristics of three distinct castor bean accessions: Hale, Rc039, and WT05. Hale is a widely cultivated variety known for its high oil yield and commercial importance (Chan et al., 2010). Rc039 is a wild accession collected from Ethiopia, while WT05 is a wild accession from Kenya. Previous research has indicated that the Ethiopian and Kenyan wild populations exhibit substantial genetic differentiation from each other and from the cultivated varieties. According to the referenced study (Xu et al., 2021), the Ethiopian and Kenyan populations have diverged due to geographical and environmental factors, leading to distinct genetic profiles. The cultivated variety shows reduced genetic diversity compared to the wild populations, likely a result of selective breeding for desirable agricultural traits. This divergence is marked by significant differences in allele frequencies and genetic structure, highlighting the impact of both natural selection and human-mediated selection on castor bean evolution (Xu et al., 2021).

By comparing these accessions, we aim to explore the distribution and evolution of TEs across both wild and cultivated castor bean genomes. The differences between these accessions could provide valuable insights into the role of TEs in genetic diversity and adaptation.

## Materials and methods

### Genome data

Genomes of WT05 and Hale were downloaded from the projects of PRJNA838012 (Lu et al., 2022) and PRJNA16585 (Chan et al., 2010) in the National Center for Biotechnology Information (NCBI) database, respectively. The Rc039 genome was downloaded from the Oil Plants database (http://oilplants.iflora.cn). As available at scaffold ordering, Hale genome was assembled into the chromosomal level by RagTag (Alonge et al., 2019; Alonge et al., 2022) using Rc039 and WT05 as the reference genome, respectively. The Hale genome assembly was further evaluated using QUAST (version 5.2.0) (Gurevich et al., 2013) and BUSCO (version 5.7.1) (Simão et al., 2015) to assess its quality and completeness. Standard metrics such as N50, L50, number of contigs/scaffolds, largest contig/scaffold, GC content, and BUSCO scores were calculated to provide a comprehensive assessment of the assembly. These results are summarized in **Supplementary Table S1**. Additionally, the completely assembled Hale genome has been compressed and made available as **Supplementary File 2**.

### Genome comparison and collinearity analysis

To assess the genomic collinearity and identify paralogous genes between the Rc039 and WT05 assemblies of castor bean, we performed all-against-all best reciprocal BLASTP searches. Paralogous genes were identified, and collinear blocks across the genomes were constructed using MCScanX (https://github.com/wyp1125/MCScanX) (Wang et al., 2012). Each block contains at least five paralogous genes. The original sequences of Chr2–5, 7, 9, and 10 of WT05 were reversely complemented. The chromosome cohort of WT05 was reordered according to the collinearity with Rc039. SubPhaser (version 1.1) (Jia et al., 2022) was used to cluster the 15-mer sequences in the castor bean genome. This analysis focuses on paralogous genes because WT05 and Rc039 are assemblies from the same species, and therefore the comparison aims to reveal gene duplication events, genomic rearrangements, and structural variations.

Orthologous genes among *Mercurialis annua*, *Manihot esculenta*, and castor bean were identified using OrthoFinder2 (version 2.2.7) (Emms and Kelly, 2019) with the parameter -S diamond. Subsequently, all single-copy orthologs were subjected to multiple sequence alignment using MAFFT (version 7.407) (Katoh and Standley, 2013), and the poorly conserved blocks were trimmed using trimAl (Capella-Gutiérrez et al., 2009) with default parameters. Finally, the consensus sequence was merged into a supergene. The phylogenetic tree was constructed using RAxML (version 8.1.2) (Stamatakis, 2014) with 100 bootstrap replicates and PROTGAMMAAUTO model and visualized using iTOL (Letunic and Bork, 2021). Protein sequences of *Mercurialis annua* and *Manihot esculenta* were downloaded from the projects of PRJEB52246 and PRJNA234389 in the National Center for Biotechnology Information (NCBI) database, respectively.

### Annotation of repeat sequences

We built a TE library of the castor bean genome using RepeatModeler (version 2.0.3) (Flynn et al., 2020) in a *de novo* approach. For this purpose, we first integrated the genome assemblies of Hale, Rc039, and WT05 into a single comprehensive assembly. This integrated assembly provided a robust basis for TE identification across different castor bean accessions. Our initial step involved running RepeatModeler on the integrated genome assembly, which generated 1,742 consensus sequences. We meticulously followed the procedural guidelines outlined in the relevant literature (Goubert et al., 2022) to scrutinize and identify the TE library generated by RepeatModeler. Our initial step involved the compilation of a prioritized list of candidates. The second step is the manual curation process. The detailed steps for the manual curation of a family are described as follows: (A) Selection of query sequence: Begin by selecting a query sequence to initiate the manual curation process; (B) BLAST search: Utilize the putative TE sequence as a query in a BLAST search against the reference genome. Record the obtained hits; (C) Extension of hit coordinates: As the prospective TE families often represent truncated versions of the actual TE, extend the hit coordinates in the genome by a specified number of bases both upstream and downstream. This ensures capturing as much TE sequence as possible; (D) Genomic sequence extraction: Extract the genomic sequences corresponding to the extended coordinates using “bedtools getfasta” (Quinlan and Hall, 2010). Subsequently, generate a multiple sequence alignment (MSA) and save it to a file; (E) Manual curation and consensus generation: Visualize the MSA in an alignment viewer and perform manual curation to generate a consensus sequence. Utilize the “cons” function from the EMBOSS (Rice et al., 2000) package to produce a consensus sequence from the MSA.

We employed TEClass (version 2.0.3) (Bickmann et al., 2023) and DeepTE (Yan et al., 2020) tools to identify TEs in the “unknown” sequences classified by RepeatModeler. Although TEClass is not yet published, we chose this tool for its promising performance in preliminary tests, which showed high accuracy in identifying various TE types through its efficient machine learning algorithm. To ensure the reliability of our results, we also conducted cross-confirmation using DeepTE. For TEClass, we set a threshold of 0.7 to increase the classification confidence. The parameters used for DeepTE were “-m P -sp P” for plant TE models, as described in its respective study. Our analysis of the RepeatModeler results revealed 353 “unknown” sequences. After reclassification using DeepTE, 266 of these sequences were identified as other TE families. Using TEClass2.0, 134 sequences were reclassified. Comparing the results of both tools, we found 103 sequences reclassified by both, with 70 sequences identified as the same TE class by both tools (**Supplementary Table S2**). The results are tabulated in **Supplementary Table S3**. A total of 134 TEs of the unknown family were re-identified, culminating in 462 consensus sequences (**Supplementary Table S4**).

We followed the naming conventions suggested in the literature (Goubert et al., 2022) for renaming TEs. One method, similar to that used by the TE repository Repbase (Bao et al., 2015), is to use the format “superfamilyX_yYyy”, where X is a unique number and yYyy is a four-letter identifier for the species in question—for example, two Ty3-retrotransposons elements from the genome of the castor bean *Ricinus communis* could be named Ty3RT-1_rCom and Ty3RT-2_ rCom, respectively. Finally, we annotated TE copies by searching the library using RepeatMasker (version 4.1.2) (Chen, 2004) with the parameters -e rmblast -cutoff 250 -xsmall -s -gff, and then we used RepeatCraft (Wong and Simakov, 2019) for post-processing the RepeatMasker annotations to generate less fragmented copies. Additionally, the curated consensus sequences and the TE annotations for each assembly are included as **Supplementary Files**. These files are compressed into a single archive named **Supplementary File 3.zip**, which contains the following: Curated_Consensus_Sequences.fasta, Hale_TE_Annotations.gff, Rc039_TE_Annotations.gff, and WT05_TE_Annotations.gff.

The structure of a full-length LTR-RT with LTR comprised the following, a pair of dinucleotide palindromic motifs flanking each LTR, the internal region including protein-coding sequences for gag, pol, and env, and a 5-bp target site duplication (TSD) flanking the element. LTRharvest (Ellinghaus et al., 2008) and LTR_FINDER (Xu and Wang, 2007) were used to identify flLTR-RTs in the castor bean genome. LTR_retriever (Ou and Jiang, 2018) was used to integrate the results of the two methods, and the flLTR-RT’s insertion time was calculated. The nucleotide mutation rate was set as *μ* = 6.9 * 10^−9^ (Ou and Jiang, 2018; Xu et al., 2021). Parameters were set as “gt ltrharvest -similar 90 -vic 10-seed 20 -seqids yes -minlenltr 100 -maxlenltr 7000 -mintsd 4 -maxtsd 6 -motif TGCA -motifmis 1” and “ltr_finder -D 15000 -d 1000 -L 7000 -l 100 -p 20 -C -M 0.9”.

We further analyzed the flLTR-RT sequences identified in the three genome assemblies. We performed clustering of the flLTR-RT sequences from the Hale, Rc039, and WT05 assemblies using CD-HIT (Fu et al., 2012). Each cluster’s representative sequence was selected, resulting in a total of 1,381 representative sequences. These representative sequences were then compared against the consensus sequences identified by RepeatModeler using BLAST+ (version 2.2.31) (Camacho et al., 2009). The BLAST+ parameters were set to a similarity threshold of 80% and a coverage threshold of 80%. This analysis revealed that 896 out of the 1,381 representative flLTR-RT sequences overlapped with the consensus sequences. The files castor_ltr.cluster, blast_results.txt, analyze_blast_results.py, and overlapping_sequences.txt were compressed into a single archive as **Supplementary File 4.zip**.

### Phylogenetic analysis of flLTR-RTs and TE divergence distribution

The identified flLTR-RT was clustered using vmatch dbcluster (Kurtz, 2003) with the parameter setting “-dbcluster 80 80 -identity 80 -exdrop 5 -seedlength 15 -d”. The genomic specificity of a cluster was defined by a decision tree algorithm: (1) if ≥90% members in a cluster are from a genome, then this cluster is assigned to this genome; (2) if less than 10% members in a cluster are from a genome, then this cluster is assigned to the shared cluster by another genome; (3) the remaining clusters are assigned to a common cluster of all genomes. Full-length LTR-RT sequences were queried against the CDD database of NCBI (Marchler-Bauer et al., 2015; Lu et al., 2020) to identify the reverse transcriptase domain. Full-length LTR-RTs of the Copia superfamily sequences from each genome were clustered using CD-HIT (Fu et al., 2012) and then were aligned using MAFFT (Katoh and Standley, 2013). Phylogenetic trees were constructed using IQ-TREE2 (Minh et al., 2020) and visualized using iTOL (Letunic and Bork, 2021).

Kimura distances between genome copies and TE consensus from the library were determined using buildSummary.pl, calcDivergenceFromAlign.pl, and createRepeatLandscape.pl (in RepeatMasker utility directory) on alignment files (.align files) after genome masking.

## Results

### Genome-wide identification of TEs in three castor bean lineages

**Figure 1:**
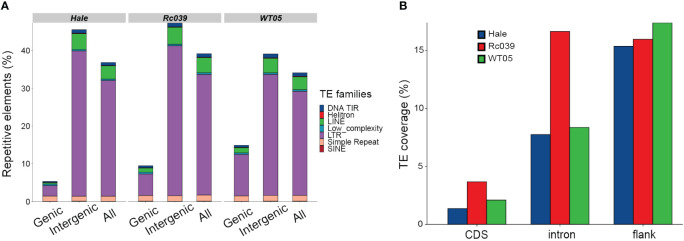
Repetitive elements distribution and relationships. **(A)** Distributions of repetitive elements in genic, intergenic, and overall genomic sequences. DNA TIR, DNA transposons characterized by terminal inverted repeats, ranging from 6 bp to several kilobases; LINE, long interspersed nuclear elements; Low_complexity, regions of simple sequence composition, often AT-rich; and SINE, short interspersed elements, which are LINE-dependent and contain internal promoters. **(B)** Proportions of TEs across different gene-associated regions: immediate 500 base-pair flanking sequences (both 5′ and 3′), introns, and coding sequences (CDS).

TEs significantly shape the genomic landscape of the three castor bean lineages studied. To comprehend the distribution and composition of transposable elements (TEs) within these lineages, we generated reference libraries using both *de novo* and homology-based approaches. Subsequently, we conducted a thorough manual inspection of these libraries. Upon detailed annotation of the individual genomes using our comprehensive TE library, we observed that the overall repeat sequence content is relatively consistent across the genomes of Rc039, WT05, and Hale, accounting for 39.17%, 34.10%, and 36.83% of the genomic sequences, respectively (**Figure 1A**). However, it is important to note that this overall consistency does not preclude variations in TE distribution within specific genomic compartments. Such variations, particularly the higher density of TEs in Rc039’s intron regions as shown in **Figure 1B**, complement the overall genomic TE content pattern, demonstrating the heterogeneous distribution of TEs across different genomic regions. The composition of most TE families is likewise congruent among these genomes—for example, of the 462 identified families, 447 (97%) are present in similar proportions in the three genomes, i.e., we found less than a twofold change of the proportion between genomes (**Supplementary Table S4**). A total of 10 most abundant families account for 20% of the total transposable elements (**Supplementary Figure S1**), consistent with other findings in wheat (Wicker et al., 2018) that a few families contribute to the vast majority of the copy number of TEs. The TEs are mainly distributed in intergenic or noncoding regions. Even at gene loci and their immediate surrounding regions, TEs distributed bias to noncoding regions, such as introns and gene flanking regions (**Figure 1B**).

In the TE landscape, a few families notably dominate in terms of copy number. Specifically, 26 families each contain over 1,500 copies, whereas a substantial number of TE families each have fewer than 500 copies. As an example, when evaluating at the superfamily level for Copia, 10 out of the 110 subfamilies hold 20,853 copies, which represents 57% of all the copies in this superfamily. A parallel pattern is found in the hAT-Ac superfamilies, where three subfamilies, making up 17% of the total, encompass 48% of all hAT-Ac copies.

### Identification and evolution of full-length LTR-RTs in the castor bean genome

LTRharvest (Ellinghaus et al., 2008) and LTR_FINDER (Xu and Wang, 2007) were used to identify flLTR-RTs in the castor bean genome. LTR_retriever (Ou and Jiang, 2018) was used to integrate the results of the two methods, and the flLTR-RT’s insertion time was calculated. **Table 1** provides a comprehensive summary of the genome assemblies and flLTR-RTs identified in the castor bean genomes, including details such as genome size, genome completeness (BUSCO values), scaffold N50, and the number of flLTR-RTs. The data in **Table 1** show that the Hale genome has significantly fewer flLTR-RTs compared to the Rc039 and WT05 genomes. This difference strongly correlates with genome assembly quality metrics such as N50 and genome completeness (BUSCO values). A multiple linear regression analysis revealed that N50 is a significant predictor of the number of flLTR-RTs (*p* = 0.036), indicating that higher N50 values are associated with a greater number of flLTR-RTs. Although the BUSCO values also showed a positive relationship, it was not statistically significant (*p* = 0.470). Specifically, Hale exhibits the smallest N50 at 0.50 Mb and a BUSCO completeness of 93%, suggesting a lower assembly quality which likely contributes to the reduced identification of flLTR-RTs. In contrast, Rc039, with the highest N50 of 32.06 Mb and a BUSCO completeness of 98%, shows the highest abundance of flLTR-RTs. This correlation suggests that better assembly quality facilitates a more comprehensive and accurate identification of flLTR-RTs, particularly evident in the Ty3-retrotransposons and unknown superfamilies.

**Table 1:** Summary of genome assemblies and flLTR-RTs in the three castor bean genomes.

	Rc039		WT05	Hale
Genome size	336 Mb		316 Mb	350 Mb
Genome completeness (complete BUSCOs)	98%		95%	93%
N50 of scaffold	32.06 Mb		31.93 Mb	0.50 Mb
TE component	39.17%		34.10%	36.83%
Copia	469		417	349
Ty3-retrotransposons	656	450	255	
Unknown	440	332	203	

We also compared the length distribution of flLTR-RTs in the three genomes (**Supplementary Figure S2**). In the Ty3-retrotransposons superfamily, the Hale genome had fewer flLTR-RTs ranging from 10,000 bp to 15,000 bp (38) compared to Rc039 (105) and WT05 (87). A Kruskal–Wallis rank sum test was conducted to compare the length distribution of flLTR-RTs among the three genomes for both the Copia and Ty3-retrotransposons superfamilies. For the Copia superfamily, whose lengths range from 4,000 bp to 5,500 bp, the test indicated no significant difference in the length distribution among the three genomes (Kruskal–Wallis chi-squared = 0.267, *df* = 2, *p*-value = 0.875). However, for the Ty3-retrotransposons superfamily, the test indicated a significant difference in the length distribution among the three genomes (Kruskal–Wallis chi-squared = 19.54, *df* = 2, *p*-value = 5.715e-05), suggesting that the assembly quality of the Hale genome may have led to fewer flLTR-RTs in the Ty3-retrotransposons superfamily.

Additionally, a Kruskal–Wallis rank sum test was conducted to compare the length distribution of TE copies among the three genomes, indicating no significant difference (Kruskal–Wallis chi-squared = 2.1313, *df* = 2, *p*-value = 0.3445) (**Supplementary Figure S2C**). Therefore, the assembly quality of the Hale genome may not significantly impact the TE copy annotation results compared to the other two castor genomes.

**Figure 2:**
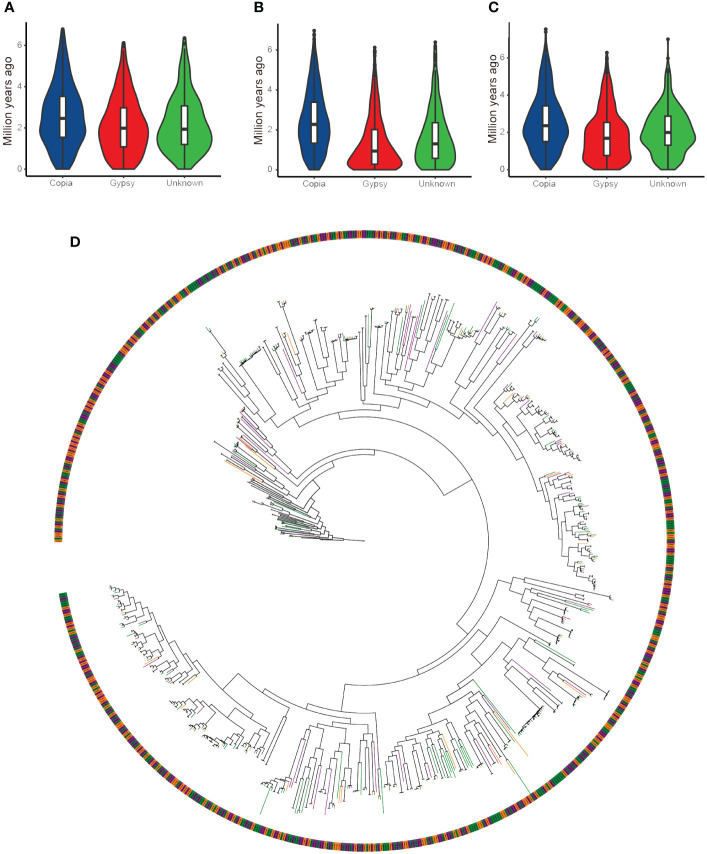
Insertion time distribution and phylogenetic tree of full-length LTR retrotransposons from the Copia superfamilies in castor bean genomes. **(A)** Insertion times in the Hale genome. **(B)** Insertion times in the WT05 genome. **(C)** Insertion times in the Rc039 genome. **(D)** Phylogenetic tree of full-length LTR retrotransposons of the Copia superfamily, color-coded by genome origin: orange branches represent the Hale genome, purple branches represent the Rc039 genome, and green branches represent the WT05 genome.

In the castor bean genome, the distribution of insertion times of flLTR-RTs belonging to the Copia superfamily was similar: the expansion started six million years ago and continued until about two million years ago (**Figures 2A–C**). The distribution of insertion time of flLTR-RTs belonging to the Ty3-retrotransposons superfamily in the three genomes was significantly different. In Rc039 and WT05, the expansion activity has continued to the present. In Hale, however, the peak of the expansion activity occurred two million years ago.

To further explore the evolutionary history of full-length LTR retrotransposons in the three genomes, we performed a phylogenetic analysis (**Figure 2D**). This phylogenetic tree demonstrates that nearly every branch contains full-length LTR retrotransposons of the Copia superfamily from all three genomes, with no genome-specific clustering. This suggests that these retrotransposons have followed similar evolutionary paths across the three genomes. Additionally, a small number of them are clustered at the basal branches of the tree, while the rest of the tree consists of clades with more branches. This evolutionary pattern is consistent with the earlier analyses of insertion times. The presence of numerous closely related branches in the terminal clades further supports the recent amplification events of LTR retrotransposons. Although the assembly level of the Hale genome was inferior to that of the other two genomes, the results of the length distribution and insertion time distribution of the full-length LTR retrotransposons showed that this did not affect the Copia superfamily.

### Different evolutionary trends between flLTR-RTs and genes

**Figure 3:**
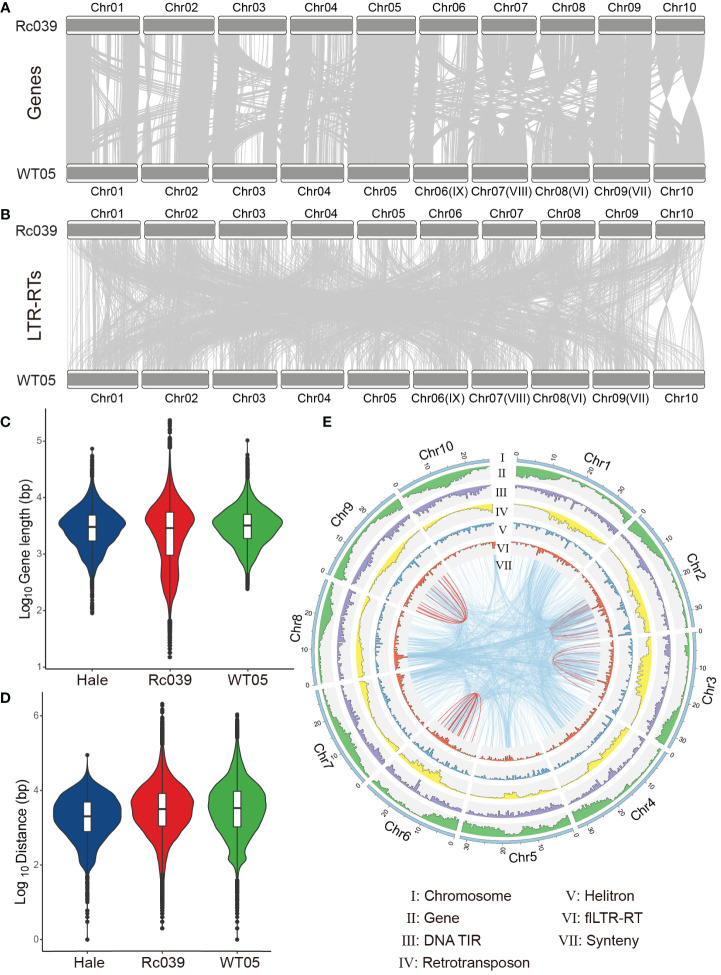
Exploring genomic relationships in castor bean varieties. **(A, B)** Genomic collinearity of genes and flLTR-RTs between the Rc039 and WT05 varieties. The light gray connectors show blocks of collinear genes. Notably, the naming convention for WT05 chromosomes (e.g., Chr#) is based on their homologous relationship with Rc039 chromosomes. The original chromosome names for WT05 are also provided in parentheses for reference. **(C, D)** Distributions of gene lengths and distances between genes, respectively, across three castor bean varieties: Hale, Rc039, and WT05. **(E)** Genomic landscape for WT05. The outermost circle represents the pseudochromosomes of WT05. The subsequent inner circles, from the exterior to the interior, showcase the genomic densities of genes, DNA TIR transposons, retrotransposons, Helitrons, and flLTR-RTs. The very innermost circle represents the synteny of flLTR-RTs—both within individual chromosomes (depicted with red lines) and between different chromosomes (shown as blue lines). An analogous landscape for the Rc039 genome can be found in **Supplementary Figure S3**.

Upon examining the genomes of the Ethiopian accession (Rc039) and the Kenyan accession (WT05) of the castor bean, we observed a pronounced chromosomal collinearity for coding genes (**Figure 3A**). Both of these genomes were sequenced and assembled independently with high precision at the chromosomal level (Xu et al., 2021; Lu et al., 2022). Local synteny gene blocks were observed between the chromosomes of Rc039 and WT05, encompassing 32,152 paralogous genes, constituting 69.4% of all identified high-confidence genes, and totaling 46,325. The conservation level of homologous chromosomes between Rc039 and WT05 surpasses that of non-homologous chromosomes. Moreover, the similarities extend to gene length and intergenic distances, which remain consistent not only between Rc039 and WT05 but also in the inbred cultivar accession, Hale (**Figures 3C, D**).

Unlike gene collinearity, flLTR-RTs exhibit reduced collinearity between homologous chromosomes across varied genomes compared to non-homologous chromosomes (**Figure 3B**). Notably, flLTR-RT’s movements between chromosomes are more prevalent than those within a single chromosome (**Figure 3E**—VII and **Supplementary Figure S3**).

We evaluated the dynamics of flLTR-RTs and genes, considering the collinearity between different chromosomes. While our initial analysis suggested a weak correlation between the gene collinearity matrix (**Figure 3A** and **Supplementary Table S5**) and the flLTR-RT collinearity matrix (**Figure 3B** and **Supplementary Table S6**), as indicated by a Spearman’s rho of 0.06 (Mantel test, *p* = 0.32), a further examination revealed a more nuanced relationship. Although the main patterns of gene and flLTR-RT collinearity are broadly conserved, with identical inversions observed in both as exemplified by Chr10, the proportion of collinear gene pairs across different chromosomes is 20.3%, which contrasts with a notably higher proportion of 58% for flLTR-RTs. This marked difference underscores the dynamic inter-chromosomal activity of flLTR-RTs, which is likely due to their active transposition and a higher frequency of insertion events compared to inter-chromosomal gene exchanges. Consequently, while the primary patterns of collinearity are conserved, the flLTR-RTs show an enhanced tendency for inter-chromosomal rearrangements, suggesting that their evolutionary trajectories exhibit distinct dynamics due to the influence of active transposable elements.

Furthermore, TEs exhibit an increasing density from the distal regions toward the centromere on the chromosome (**Figure 3E**–I–VI). In contrast, genes demonstrate the reverse trend. Within the WT05 genome, the distribution pattern of most TEs is inversely related to that of genes, for instance, retrotransposons show a negative correlation with gene distribution on chromosomes (rho = −0.15, *p* < 0.01).

### Cultivar Hale may be derived from the Ethiopia lineage

The origins of the Hale breed may be traced to the Ethiopian Rc039 lineage. We reassembled Hale’s scaffolds to the chromosome scale using Ethiopian Rc039 and Kenyan WT05, respectively, as reference genomes. The different k-mers with a range from 5 to 51 were used to test their potential effects on the different genomes. With the exception of the 5-mers and the 7-mers, which lacked sufficient differential k-mers for phasing, all other k-mer lengths were able to phase all three genomes perfectly. The number of the identified differential k-mers stabilized at 15-mers is close to the conclusions reached by previous related studies (Jia et al., 2022).

**Figure 4:**
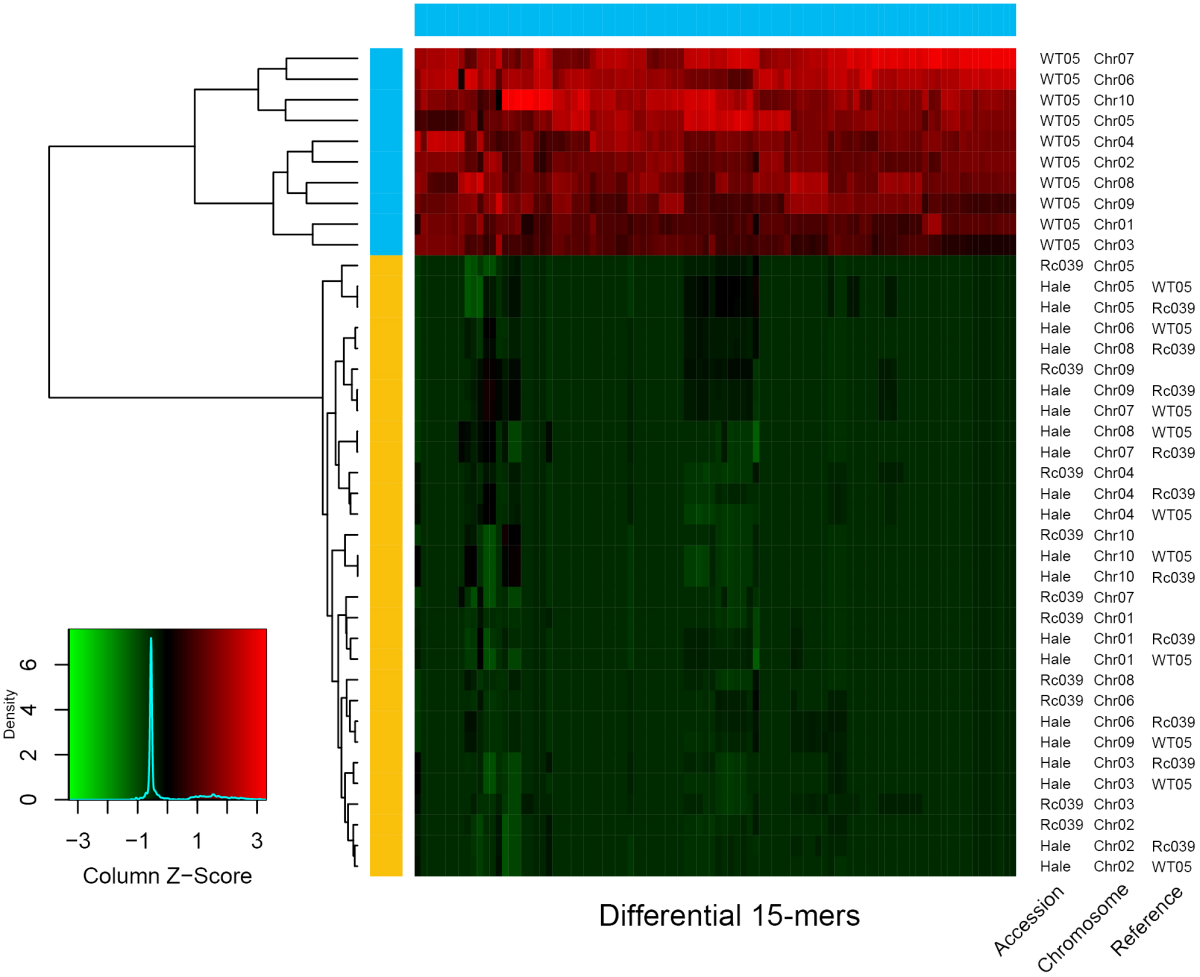
Relationship of Ethiopia (Rc039), Kenya (WT05), and cultivar accession (Hale). The horizontal color bar at the top of the heat map indicates in which chromosome set the 15-mer is differentially abundant; the vertical color bar on the left indicates the chromosome set to which the chromosome is assigned. The heat map indicates the Z-scaled relative abundance of 15-mers. The larger the Z score, the higher the relative abundance of a 15-mer. As available at the scaffold level, Hale genome was assembled into chromosomal level using Rc039 and WT05 as reference genome, respectively. The tree on the left is a clustering tree. The plots were produced using SubPhaser (Jia et al., 2022).

In **Figure 4**, four genomes are represented: Rc039, WT05, and the chromosome-level Hale genome assembled using these two genomes as templates. It can be seen from the clustering tree in **Figure 4** that the k-mers belonging to WT05 cluster together, while the k-mers belonging to Rc039 and the two Hale genomes cluster together. The k-mers of WT05 exhibit clear distinctions from the other three genomes, while the differential k-mers in the two Hale genomes are notably more similar to those in Rc039. Hale consistently demonstrated stronger genetic ratios with Rc039 than with WT05 (**Figure 4**). These findings suggest that the domestication of Hale may be related to the ancestor of Rc039, although further phylogenomic analysis based on host genes is required to confirm this evolutionary relationship.

**Figure 5:**
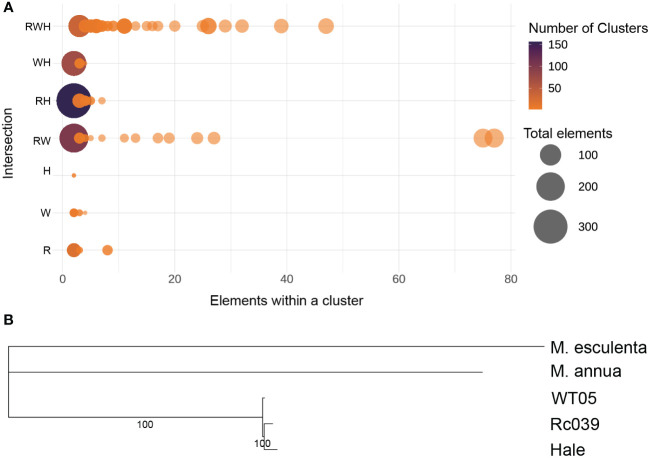
Intersection of transposable element. **(A)** Intersection of flLTR-RTs across castor bean genomes. R, Rc039; W, WT05; H, Hale. Three and two letters represent the intersection of three and two genomes, respectively. A single letter represents an accession-specific set. X-axis, cluster size; the color coding gives the number of clusters; the circle area corresponds to the number of elements. The circle on the left represents the largest area, indicating that the majority of full-length LTR retrotransposons are not concentrated within a single cluster. **(B)** Phylogenetic tree including *Mercurialis annua*, *Manihot esculenta*, and the three castor bean genomes.

We analyzed 3,571 full-length LTR retrotransposons in castor bean genomes, grouping them based on sequence similarity. Using an 80/80 cluster criterion (80% sequence identity and 80% coverage) (Wicker et al., 2007), we identified 78 families common to Rc039, WT05, and Hale (**Figure 5A**). The phylogenetic trees, constructed using sequences from the three major shared families, reveal that these genomes are closely intertwined, pointing to a shared, undifferentiated ancestor (**Supplementary Figure S4**). We analyzed the proportions of flLTR-RT families shared among the Rc039, WT05, and Hale genomes. We found that the correlation of the proportions of flLTR-RT families within each genome was high, with a Pearson correlation coefficient of 0.98 between each genome pair (*p* < 0.05), indicating statistically significant similarities (**Supplementary Table S7**). Further pairwise comparisons showed a correlation of 0.96 between WT05 and Hale for the proportions of shared flLTR-RT families within these genomes (**Supplementary Table S8**) and a correlation of 0.97 between Rc039 and Hale for the proportions of shared flLTR-RT families within these genomes (**Supplementary Table S9**, Pearson test, *p* < 0.05), suggesting strong and statistically significant similarities among these genomes’ flLTR-RT families. Our analysis also revealed that within the same subfamily of flLTR-RTs, not only is there a close evolutionary relationship but also the proportion within the genomes is similar.

To determine the evolutionary relationship among the accessions, we performed a phylogenomic analysis based on orthologous genes (**Figure 5B**). This analysis included *Mercurialis annua*, *Manihot esculenta*, and the three castor bean genomes (Hale, Rc039, and WT05). The results show that Rc039 and Hale are the closest in evolutionary terms, indicating a closer evolutionary relationship between these two genomes. This supports our hypothesis that Hale may have been domesticated from an ancestral population related to Rc039.

### TE dynamics in castor bean genomes

**Figure 6:**
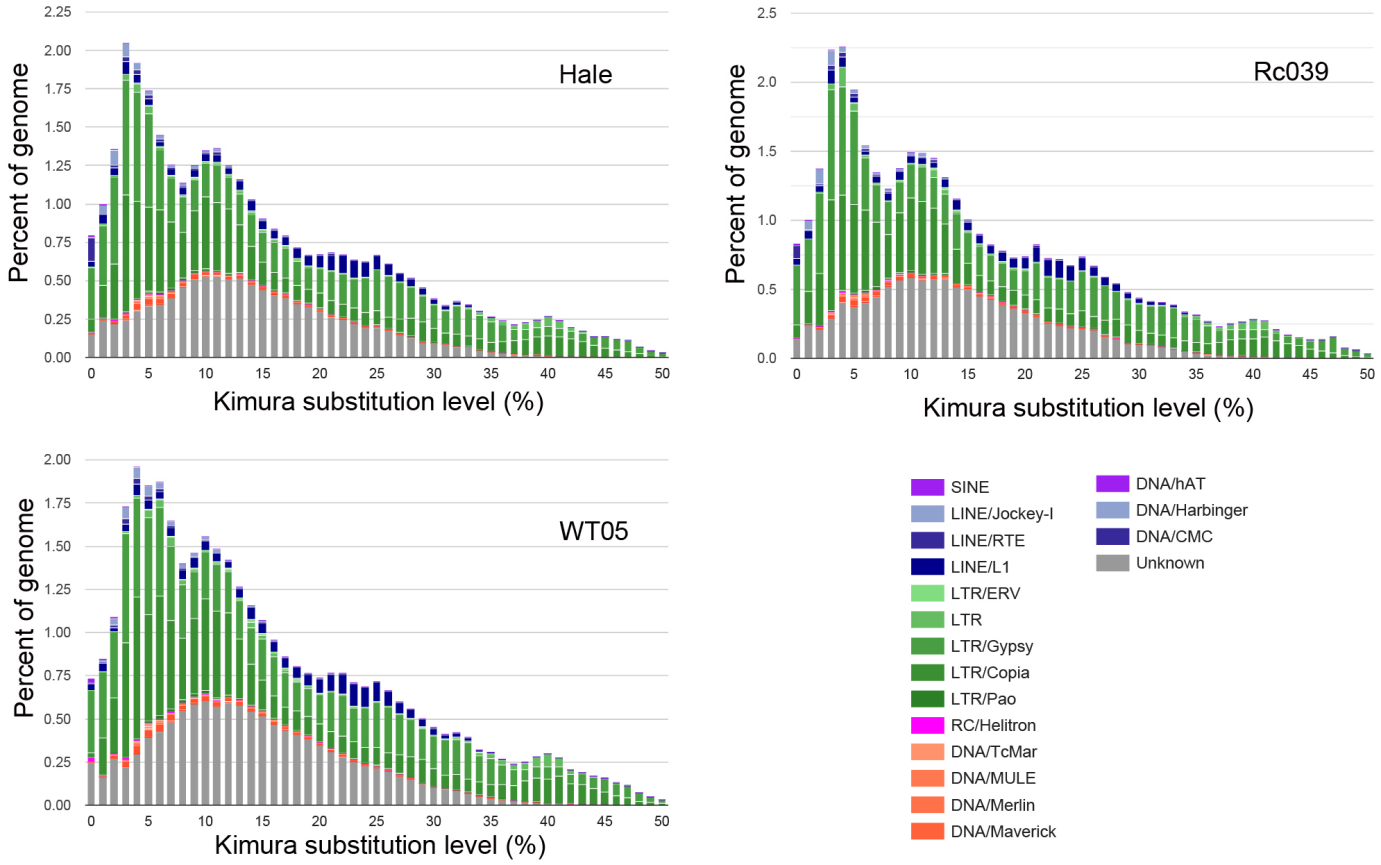
Kimura distance-based copy divergence analyses of TE in castor bean. TEs are classified according to their *K* (Kimura) values. Clustering was performed according to their Kimura distances (K-value from 0 to 50). Copies clustering on the left side of the graph did not greatly diverge from the consensus sequence and potentially corresponded to recent events, while sequences on the right side likely corresponded to older divergence.

To infer transposition times, TEs are classified according to their *K* (Kimura) values. The extent of sequence alignment between a TE copy and its counterpart in the TE reference library sheds light on the timing of its transposition. Copies showing a significant similarity (*K* < 25) indicate a recent transposition activity, whereas copies with a reduced similarity (*K* > 25) suggest more distant transposition events (**Figure 6**) (Chalopin et al., 2015).

The castor bean genome has experienced multiple TE activity bursts. The first notable burst, centered on *K* = 10, is marked by a significant increase in the unknown superfamily, while the other superfamilies remain comparatively unchanged. Following this, additional activity bursts are observed, including a prominent surge near *K* = 5, predominantly characterized by the expansion of the Ty3-retrotransposons superfamily. This consistent growth of the Ty3-retrotransposons superfamily aligns with the evolutionary patterns seen in flLTR-RTs within the castor bean genome. As shown in **Figure 6**, there appear to be four distinct TE activity bursts in total.

## Discussion

We analyzed the TE landscape in castor bean accessions, examining aspects such as abundance, diversity, activity, and evolutionary history. Our findings reveal differences in the behavior and dynamics of TEs compared to coding genes. While the chromosome structure remains relatively stable in terms of gene order across different accessions, indicating limited genetic differences, TEs play a pivotal role in two significant evolutionary processes for castor bean: the temporal dynamics of evolution and the generation of genetic variability.

Our findings reveal a remarkable consistency in the distribution and composition of TEs across the three studied lineages, collectively constituting a substantial portion of their respective genomes. The presence of certain TE families with significantly higher copy numbers underscores their pronounced influence on the genomic architecture of castor bean lineages.

TEs are widely acknowledged as pivotal drivers of genome expansion (Kidwell, 2002; Gao et al., 2016; Pellicer et al., 2018). Within plant genomes, retrotransposons, notably LTR transposons, represent the predominant category of TEs (Jiménez-Ruiz et al., 2020; Zavallo et al., 2020; Wang et al., 2021). Our investigation unveils the distinctive contributions of various superfamilies to the expansion of the three castor bean genomes. These dynamics in the genome’s superfamily composition are shaped by the ongoing processes of superfamilies’ loss and retention during species evolution. The interplay between the retention and loss of TEs in the host genome assumes paramount importance, further underscored by the regulatory role of genomic defense mechanisms like DNA methylation (Levin and Moran, 2011).

As a cultivar, compared with the long evolutionary history, the short domestication history of Hale does not seem to affect the distribution of flLTR-RT’s insertion time so obviously. Combined with the length distribution of flLTR-RTs in the Ty3-retrotransposons superfamily, we believe that in the Hale genome, a part of recently amplified flLTR-RTs belonging to the Ty3-retrotransposons superfamily has not been identified. The current flLTR-RTs content is the outcome of two opposing forces: insertion and removal (Wicker et al., 2018). In the WT05 genome, the number of young flLTR-RTs continued to accumulate. We suggest that counter-selection of harmful flLTR-RT insertions was stringent in the WT05 genome. The differences in the age distribution of flLTR-RTs across the three genomes suggest that genetic differentiation between castor bean genomes has occurred in places other than genes.

The comparison between TE collinearity and gene collinearity raises intriguing questions about the mechanisms governing their evolution. The weaker correlation between TE collinearity and gene collinearity implies that TEs have followed independent evolutionary paths, possibly driven by their unique modes of replication and regulation. This divergence indicates the complexity of genome evolution and suggests the need for further research into the specific mechanisms governing TE dynamics. Additionally, we recognize the potential value of investigating transposon insertion polymorphisms (TIPs) as a future research direction. We plan to utilize our existing dataset in future research to explore the impact of TEs on genome variation and their potential applications in genome-wide association studies (GWAS).

While new TEs possess the capacity to proliferate within the genome, the host genome has mechanisms to counteract such changes, restricting the expansion of TEs. Nevertheless, if the inserted TE confers benefits to the host genome, it may be preserved and undergo co-evolution with the host (Kidwell and Lisch, 2001; Hua-Van et al., 2005; Elliott, 2016). Consequently, TE bursts could be linked to significant evolutionary events, with prior research indicating a correlation between speciation and heightened TE activity (Oliver and Greene, 2011; Dion-Côté et al., 2014).

Our investigation into the genetic relationship between the Hale and Rc039 lineages provides genetic evidence implying the hypothesis that Hale may have originated from the ancestral lineage of Rc039. These results indicate that TEs contribute to evolutionary processes for castor bean: the increase of genetic variability, agreeing with other species (Jiménez-Ruiz et al., 2020; Stritt et al., 2020; Colonna Romano and Fanti, 2022). As castor bean populations have diverged significantly at the TE level, their genetic distinctiveness may be comparable to that observed in natural geographic races, such as the four diversity centers worldwide (Anjani, 2012). This insight has significant implications for understanding the domestication and lineage origins of cultivated castor bean varieties. However, our current study does not provide direct experimental evidence to fully support the influence of TEs on the genome evolution of castor bean. Further experimental validation is required to substantiate these claims.

Finally, our study provides insights into TE dynamics in castor bean genomes. These findings have implications for the understanding of plant genomics and the evolution of plant genomes in response to transposable elements. Future research in this field will likely uncover additional layers of complexity in the interaction between TEs and host genomes.

## Conclusion

In summary, our study provides an analysis of transposable elements (TEs) in three castor bean lineages—Rc039, WT05, and Hale. We have observed consistent TE distribution and composition across these lineages, implying the substantial role of TEs in shaping castor bean genomes. The comparison between TE collinearity and gene collinearity highlights the distinct dynamics of TEs compared to genes, and the genetic relationship between Hale and Rc039 indicates the significance of TEs in castor bean genetics and evolution. However, further experimental validation is needed to substantiate these findings. These insights contribute valuable knowledge to the field of plant genomics and castor bean research.

## Data availability statement

The original contributions presented in the study are included in the article/**Supplementary Material**. Further inquiries can be directed to the corresponding authors.

## Author contributions

LK: Writing – original draft, Writing – review & editing. TZ: Writing – original draft, Writing – review & editing. LM: Writing – original draft, Writing – review & editing.

## References

[B1] Alonge M. Soyk S. Ramakrishnan S. Wang X. Goodwin S. Sedlazeck F. J.. (2019). RaGOO: fast and accurate reference-guided scaffolding of draft genomes. Genome Biol. 20, 1–17. doi: 10.1186/s13059-019-1829-6 31661016 PMC6816165

[B2] Alonge M. Lebeigle L. Kirsche M. Jenike K. Ou S. Aganezov S.. (2022). Automated assembly scaffolding using RagTag elevates a new tomato system for high-throughput genome editing. Genome Biol. 23, 258. doi: 10.1186/s13059-022-02823-7 36522651 PMC9753292

[B3] Anjani K. (2012). Castor genetic resources: A primary gene pool for exploitation. Ind. Crops Products 35, 1–14. doi: 10.1016/j.indcrop.2011.06.011

[B4] Bao W. Kojima K. K. Kohany O. (2015). Repbase Update, a database of repetitive elements in eukaryotic genomes. Mobile DNA 6, 1–6. doi: 10.1186/s13100-015-0041-9 PMC4455052 26045719

[B5] Bickmann L. Rodriguez M. Jiang X. Makalowski W. (2023). TEclass2: Classification of transposable elements using Transformers. bioRxiv 2023, 2010. 2013.562246. doi: 10.1101/2023.10.13.562246

[B6] Britten R. J. Davidson E. H. (1971). Repetitive and non-repetitive DNA sequences and a speculation on the origins of evolutionary novelty. Q. Rev. Biol. 46, 111–138. doi: 10.1086/406830 5160087

[B7] Brosius J. (2019). Exaptation at the molecular genetic level. Sci. China Life Sci. 62, 437–452. doi: 10.1007/s11427-018-9447-8 30798493

[B8] Camacho C. Coulouris G. Avagyan V. Ma N. Papadopoulos J. Bealer K.. (2009). BLAST+: architecture and applications. BMC Bioinf. 10, 1–9. doi: 10.1186/1471-2105-10-421 PMC2803857 20003500

[B9] Capella-Gutiérrez S. Silla-Martínez J. M. Gabaldón T. (2009). trimAl: a tool for automated alignment trimming in large-scale phylogenetic analyses. Bioinformatics 25, 1972–1973. doi: 10.1093/bioinformatics/btp348 19505945 PMC2712344

[B10] Chalopin D. Naville M. Plard F. Galiana D. Volff J.-N. (2015). Comparative analysis of transposable elements highlights mobilome diversity and evolution in vertebrates. Genome Biol. Evol. 7, 567–580. doi: 10.1093/gbe/evv005 25577199 PMC4350176

[B11] Chan A. P. Crabtree J. Zhao Q. Lorenzi H. Orvis J. Puiu D.. (2010). Draft genome sequence of the oilseed species Ricinus communis. Nat. Biotechnol. 28, 951–956. doi: 10.1038/nbt.1674 20729833 PMC2945230

[B12] Chen N. (2004). Using Repeat Masker to identify repetitive elements in genomic sequences. Curr. Protoc. Bioinf. 5, 4.10.11–14.10. 14. doi: 10.1002/0471250953.bi0410s05 18428725

[B13] Choi J. Y. Lee Y. C. G. (2020). Double-edged sword: The evolutionary consequences of the epigenetic silencing of transposable elements. PloS Genet. 16, e1008872. doi: 10.1371/journal.pgen.1008872 32673310 PMC7365398

[B14] Choulet F. Alberti A. Theil S. Glover N. Barbe V. Daron J.. (2014). Structural and functional partitioning of bread wheat chromosome 3B. Science 345, 1249721. doi: 10.1126/science.1249721 25035497

[B15] Chuong E. B. Elde N. C. Feschotte C. (2016). Regulatory evolution of innate immunity through co-option of endogenous retroviruses. Science 351, 1083–1087. doi: 10.1126/science.aad5497 26941318 PMC4887275

[B16] Colonna Romano N. Fanti L. (2022). Transposable elements: major players in shaping genomic and evolutionary patterns. Cells 11, 1048. doi: 10.3390/cells11061048 35326499 PMC8947103

[B17] Dion-Côté A.-M. Renaut S. Normandeau E. Bernatchez L. (2014). RNA-seq reveals transcriptomic shock involving transposable elements reactivation in hybrids of young lake whitefish species. Mol. Biol. Evol. 31, 1188–1199. doi: 10.1093/molbev/msu069 24505119

[B18] Drongitis D. Aniello F. Fucci L. Donizetti A. (2019). Roles of transposable elements in the different layers of gene expression regulation. Int. J. Mol. Sci. 20, 5755. doi: 10.3390/ijms20225755 31731828 PMC6888579

[B19] Eickbush T. H. Furano A. V. (2002). Fruit flies and humans respond differently to retrotransposons. Curr. Opin. Genet. Dev. 12, 669–674. doi: 10.1016/S0959-437X(02)00359-3 12433580

[B20] Ellinghaus D. Kurtz S. Willhoeft U. (2008). LTRharvest, an efficient and flexible software for *de novo* detection of LTR retrotransposons. BMC Bioinf. 9, 1–14. doi: 10.1186/1471-2105-9-18 PMC2253517 18194517

[B21] Elliott T. A. (2016). Conceptual and empirical investigations of eukaryotic transposable element evolution (Doctoral dissertation, University of Guelph).

[B22] Emms D. M. Kelly S. (2019). OrthoFinder: phylogenetic orthology inference for comparative genomics. Genome Biol. 20, 1–14. doi: 10.1186/s13059-019-1832-y 31727128 PMC6857279

[B23] Flynn J. M. Hubley R. Goubert C. Rosen J. Clark A. G. Feschotte C.. (2020). RepeatModeler2 for automated genomic discovery of transposable element families. Proc. Natl. Acad. Sci. 117, 9451–9457. doi: 10.1073/pnas.1921046117 32300014 PMC7196820

[B24] Fu L. Niu B. Zhu Z. Wu S. Li W. (2012). CD-HIT: accelerated for clustering the next-generation sequencing data. Bioinformatics 28, 3150–3152. doi: 10.1093/bioinformatics/bts565 23060610 PMC3516142

[B25] Gao B. Shen D. Xue S. Chen C. Cui H. Song C. (2016). The contribution of transposable elements to size variations between four teleost genomes. Mobile DNA 7, 1–16. doi: 10.1186/s13100-016-0059-7 26862351 PMC4746887

[B26] Goubert C. Craig R. J. Bilat A. F. Peona V. Vogan A. A. Protasio A.V. (2022). A beginner’s guide to manual curation of transposable elements. Mobile DNA 13. doi: 10.1186/s13100-021-00259-7 PMC8969392 35354491

[B27] Gurevich A. Saveliev V. Vyahhi N. Tesler G. (2013). QUAST: quality assessment tool for genome assemblies. Bioinformatics 29, 1072–1075. doi: 10.1093/bioinformatics/btt086 23422339 PMC3624806

[B28] Horváth V. Merenciano M. González J. (2017). Revisiting the relationship between transposable elements and the eukaryotic stress response. Trends Genet. 33, 832–841. doi: 10.1016/j.tig.2017.08.007 28947157

[B29] Hua-Van A. Le Rouzic A. Maisonhaute C. Capy P. (2005). Abundance, distribution and dynamics of retrotransposable elements and transposons: similarities and differences. Cytogenetic Genome Res. 110, 426–440. doi: 10.1159/000084975 16093695

[B30] Jia K. H. Wang Z. X. Wang L. Li G. Y. Zhang W. Wang X. L.. (2022). SubPhaser: A robust allopolyploid subgenome phasing method based on subgenome-specific k-mers. New Phytol. 235, 801–809. doi: 10.1111/nph.18173 35460274

[B31] Jiménez-Ruiz J. Ramírez-Tejero J. A. Fernández-Pozo N. Leyva-Pérez M. d. l. O. Yan H. Rosa R. d. l.. (2020). Transposon activation is a major driver in the genome evolution of cultivated olive trees (Olea europaea L.). Plant Genome 13, e20010. doi: 10.1002/tpg2.20010 33016633 PMC12806974

[B32] Kallamadi P. R. Nadigatla V. G. R. Mulpuri S. (2015). Molecular diversity in castor (Ricinus communis L.). Ind. Crops products 66, 271–281. doi: 10.1016/j.indcrop.2014.12.061

[B33] Katoh K. Standley D. M. (2013). MAFFT multiple sequence alignment software version 7: improvements in performance and usability. Mol. Biol. Evol. 30, 772–780. doi: 10.1093/molbev/mst010 23329690 PMC3603318

[B34] Kidwell M. G. (2002). Transposable elements and the evolution of genome size in eukaryotes. Genetica 115, 49–63. doi: 10.1023/A:1016072014259 12188048

[B35] Kidwell M. G. Lisch D. R. (2001). Perspective: transposable elements, parasitic DNA, and genome evolution. Evolution 55, 1–24. doi: 10.1111/j.0014-3820.2001.tb01268.x 11263730

[B36] Kurtz S. (2003). The Vmatch large scale sequence analysis software. Ref Type: Comput. Program 412, 297.

[B37] Lanciano S. Mirouze M. (2018). Transposable elements: all mobile, all different, some stress responsive, some adaptive? Curr. Opin. Genet. Dev. 49, 106–114. doi: 10.1016/j.gde.2018.04.002 29705597

[B38] Letunic I. Bork P. (2021). Interactive Tree Of Life (iTOL) v5: an online tool for phylogenetic tree display and annotation. Nucleic Acids Res. 49, W293–W296. doi: 10.1093/nar/gkab301 33885785 PMC8265157

[B39] Levin H. L. Moran J. V. (2011). Dynamic interactions between transposable elements and their hosts. Nat. Rev. Genet. 12, 615–627. doi: 10.1038/nrg3030 21850042 PMC3192332

[B40] Lu J. Pan C. Fan W. Liu W. Zhao H. Li D.. (2022). A Chromosome-level Genome Assembly of Wild Castor Provides New Insights into its Adaptive Evolution in Tropical Desert. Genomics Proteomics Bioinf. 20, 42–59. doi: 10.1016/j.gpb.2021.04.003 PMC9510866 34339842

[B41] Lu S. Wang J. Chitsaz F. Derbyshire M. K. Geer R. C. Gonzales N. R.. (2020). CDD/SPARCLE: the conserved domain database in 2020. Nucleic Acids Res. 48, D265–D268. doi: 10.1093/nar/gkz991 31777944 PMC6943070

[B42] Maksakova I. A. Romanish M. T. Gagnier L. Dunn C. A. Van de Lagemaat L. N. Mager D. L. (2006). Retroviral elements and their hosts: insertional mutagenesis in the mouse germ line. PloS Genet. 2, e2. doi: 10.1371/journal.pgen.0020002 16440055 PMC1331978

[B43] Marchler-Bauer A. Derbyshire M. K. Gonzales N. R. Lu S. Chitsaz F. Geer L. Y.. (2015). CDD: NCBI's conserved domain database. Nucleic Acids Res. 43, D222–D226. doi: 10.1093/nar/gku1221 25414356 PMC4383992

[B44] Minh B. Q. Schmidt H. A. Chernomor O. Schrempf D. Woodhams M. D. Von Haeseler A.. (2020). IQ-TREE 2: new models and efficient methods for phylogenetic inference in the genomic era. Mol. Biol. Evol. 37, 1530–1534. doi: 10.1093/molbev/msaa015 32011700 PMC7182206

[B45] Oliver K. R. Greene W. K. (2011). Mobile DNA and the TE-Thrust hypothesis: supporting evidence from the primates. Mobile DNA 2, 1–17. doi: 10.1186/1759-8753-2-8 21627776 PMC3123540

[B46] Ou S. Jiang N. (2018). LTR_retriever: a highly accurate and sensitive program for identification of long terminal repeat retrotransposons. Plant Physiol. 176, 1410–1422. doi: 10.1104/pp.17.01310 29233850 PMC5813529

[B47] Pellicer J. Hidalgo O. Dodsworth S. Leitch I. J. (2018). Genome size diversity and its impact on the evolution of land plants. Genes 9, 88. doi: 10.3390/genes9020088 29443885 PMC5852584

[B48] Quinlan A. R. Hall I. M. (2010). BEDTools: a flexible suite of utilities for comparing genomic features. Bioinformatics 26, 841–842. doi: 10.1093/bioinformatics/btq033 20110278 PMC2832824

[B49] Rebollo R. Karimi M. M. Bilenky M. Gagnier L. Miceli-Royer K. Zhang Y.. (2011). Retrotransposon-induced heterochromatin spreading in the mouse revealed by insertional polymorphisms. PloS Genet. 7, e1002301. doi: 10.1371/journal.pgen.1002301 21980304 PMC3183085

[B50] Rice P. Longden I. Bleasby A. (2000). EMBOSS: the European molecular biology open software suite. Trends Genet. 16, 276–277. doi: 10.1016/S0168-9525(00)02024-2 10827456

[B51] Schnable P. S. Ware D. Fulton R. S. Stein J. C. Wei F. Pasternak S.. (2009). The B73 maize genome: complexity, diversity, and dynamics. science 326, 1112–1115. doi: 10.1126/science.1178534 19965430

[B52] Simão F. A. Waterhouse R. M. Ioannidis P. Kriventseva E. V. Zdobnov E. M. (2015). BUSCO: assessing genome assembly and annotation completeness with single-copy orthologs. Bioinformatics 31, 3210–3212. doi: 10.1093/bioinformatics/btv351 26059717

[B53] Stamatakis A. (2014). RAxML version 8: a tool for phylogenetic analysis and post-analysis of large phylogenies. Bioinformatics 30, 1312–1313. doi: 10.1093/bioinformatics/btu033 24451623 PMC3998144

[B54] Stapley J. Santure A. W. Dennis S. R. (2015). Transposable elements as agents of rapid adaptation may explain the genetic paradox of invasive species. Mol. Ecol. 24, 2241–2252. doi: 10.1111/mec.13089 25611725

[B55] Stritt C. Wyler M. Gimmi E. L. Pippel M. Roulin A. C. (2020). Diversity, dynamics and effects of long terminal repeat retrotransposons in the model grass Brachypodium distachyon. New Phytol. 227, 1736–1748. doi: 10.1111/nph.16308 31677277 PMC7497039

[B56] Sundaram V. Cheng Y. Ma Z. Li D. Xing X. Edge P.. (2014). Widespread contribution of transposable elements to the innovation of gene regulatory networks. Genome Res. 24, 1963–1976. doi: 10.1101/gr.168872.113 25319995 PMC4248313

[B57] Thatikunta R. Siva Sankar A. Sreelakshmi J. Palle G. Leela C. Durga Rani C. V.. (2016). Utilization of in silico EST–SSR markers for diversity studies in castor (Ricinus communis L.). Physiol. Mol. Biol. Plants 22, 535–545. doi: 10.1007/s12298-016-0367-x 27924126 PMC5120032

[B58] Wang Y. Tang H. DeBarry J. D. Tan X. Li J. Wang X.. (2012). MCScanX: a toolkit for detection and evolutionary analysis of gene synteny and collinearity. Nucleic Acids Res. 40, e49. doi: 10.1093/nar/gkr1293 22217600 PMC3326336

[B59] Wang M. Li J. Wang P. Liu F. Liu Z. Zhao G.. (2021). Comparative genome analyses highlight transposon-mediated genome expansion and the evolutionary architecture of 3D genomic folding in cotton. Mol. Biol. Evol. 38, 3621–3636. doi: 10.1093/molbev/msab128 33973633 PMC8382922

[B60] Wicker T. Sabot F. Hua-Van A. Bennetzen J. L. Capy P. Chalhoub B.. (2007). A unified classification system for eukaryotic transposable elements. Nat. Rev. Genet. 8, 973–982. doi: 10.1038/nrg2165 17984973

[B61] Wicker T. Gundlach H. Spannagl M. Uauy C. Borrill P. Ramírez-González R. H.. (2018). Impact of transposable elements on genome structure and evolution in bread wheat. Genome Biol. 19, 1–18. doi: 10.1186/s13059-018-1479-0 30115100 PMC6097303

[B62] Wong W. Y. Simakov O. (2019). RepeatCraft: a meta-pipeline for repetitive element de-fragmentation and annotation. Bioinformatics 35, 1051–1052. doi: 10.1093/bioinformatics/bty745 30165587 PMC6419915

[B63] Xu W. Wu D. Yang T. Sun C. Wang Z. Han B.. (2021). Genomic insights into the origin, domestication and genetic basis of agronomic traits of castor bean. Genome Biol. 22, 1–27. doi: 10.1186/s13059-021-02333-y 33874982 PMC8056531

[B64] Xu Z. Wang H. (2007). LTR_FINDER: an efficient tool for the prediction of full-length LTR retrotransposons. Nucleic Acids Res. 35, W265–W268. doi: 10.1093/nar/gkm286 17485477 PMC1933203

[B65] Yan H. Bombarely A. Li S. (2020). DeepTE: a computational method for *de novo* classification of transposons with convolutional neural network. Bioinformatics 36, 4269–4275. doi: 10.1093/bioinformatics/btaa519 32415954

[B66] Zavallo D. Crescente J. M. Gantuz M. Leone M. Vanzetti L. S. Masuelli R. W.. (2020). Genomic re-assessment of the transposable element landscape of the potato genome. Plant Cell Rep. 39, 1161–1174. doi: 10.1007/s00299-020-02554-8 32435866

