# Supplementary material for: The impact of retrotransposons on castor bean genomes
Source: Front Plant Sci. 2024 Jul 23;15:1397215. doi: 10.3389/fpls.2024.1397215 (PMC11300327; doi:10.3389/fpls.2024.1397215)
Supplement: Supplementary file 3 [file DataSheet_3.docx]

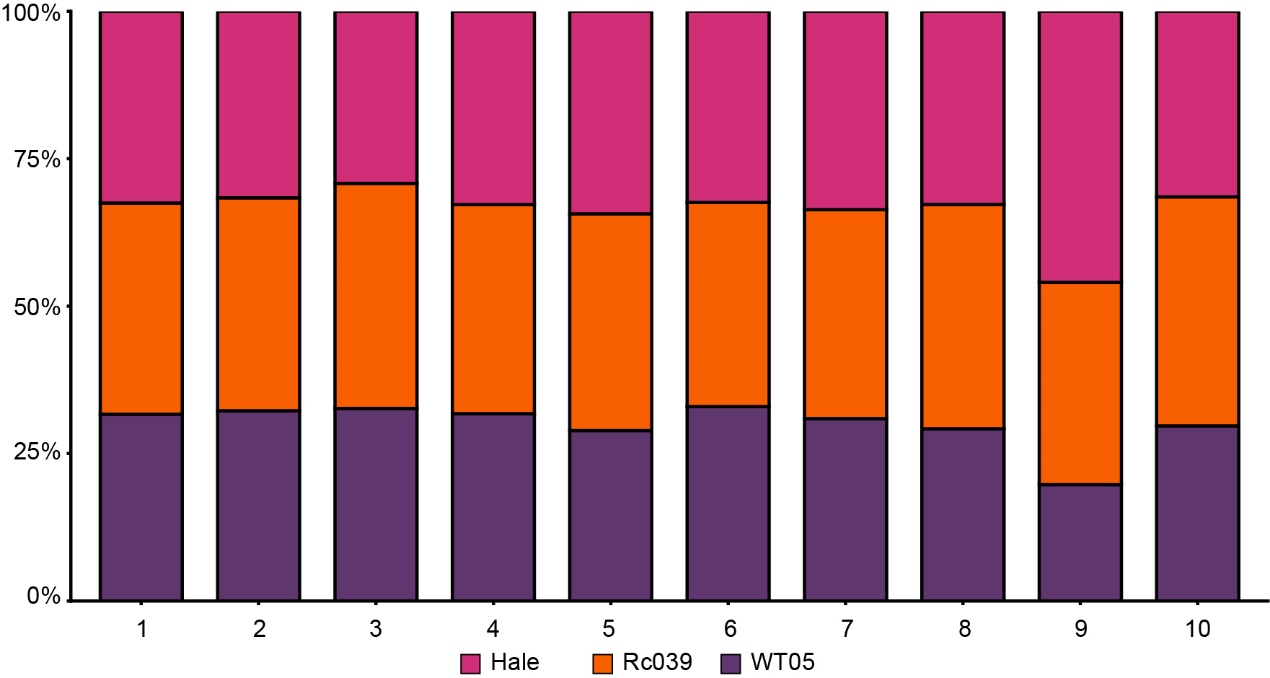


**Figure S1. Proportions of the 10 most abundant TE families.** For each family, the fractions of Hale, Rc039 and WT05 are represented in red, orange, and purple, respectively. 1, Copia-64_rCom; 2, Copia-85_rCom; 3, Ty3RT-11_rCom; 4, Ty3RT-2_rCom; 5, Ty3RT-34_rCom; 6, Ty3RT-40_rCom; 7, Ty3RT-7_rCom; 8, Ty3RT-9_rCom; 9, L1-22_rCom; 10, LTR_rCom.


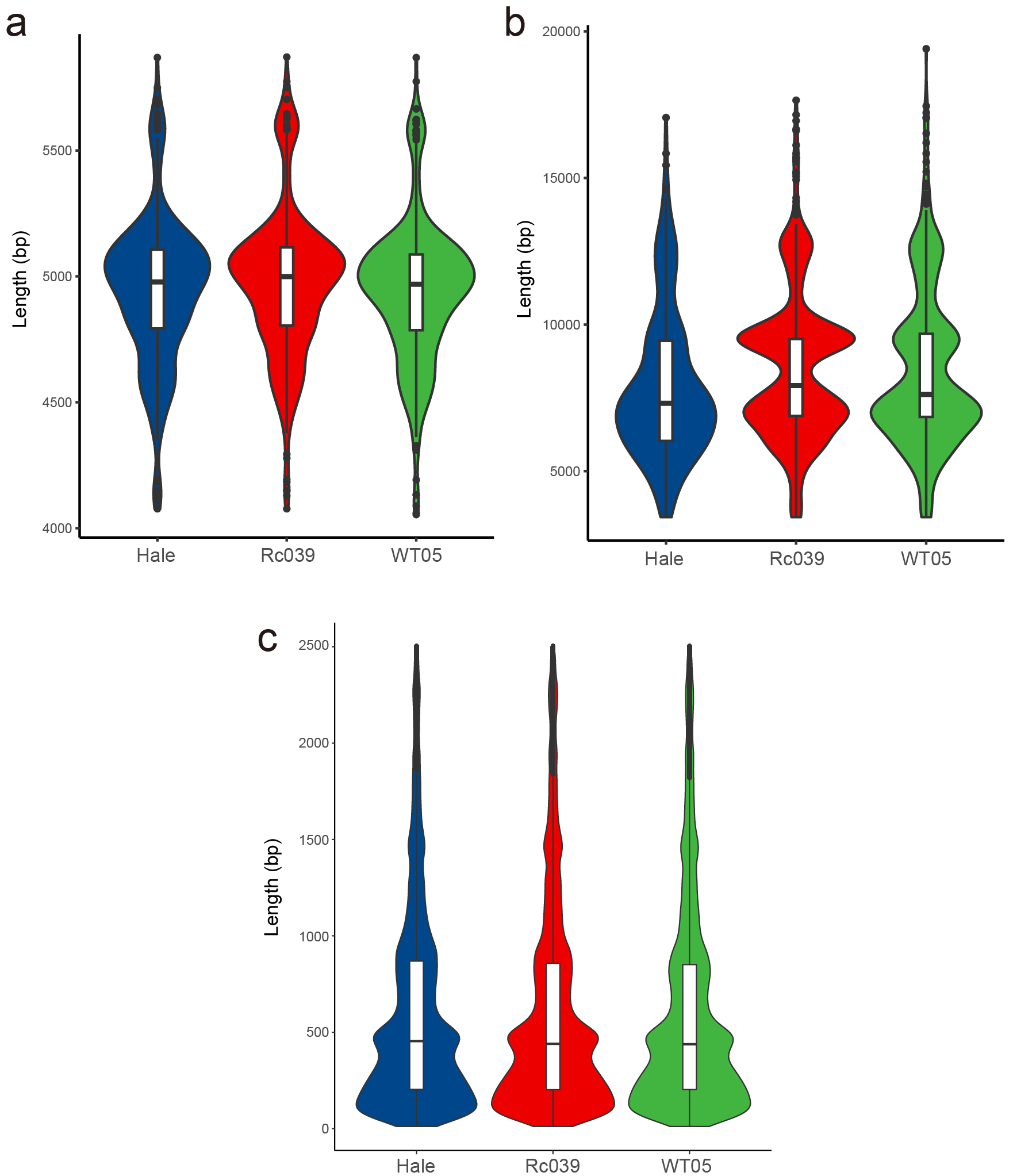


**Figure S2. Length distribution of flLTR-RTs in castor bean genomes. (a) Copia. (b) Ty3-retrotransposons. (c) Length distribution of transposon copies in the three genomes.**


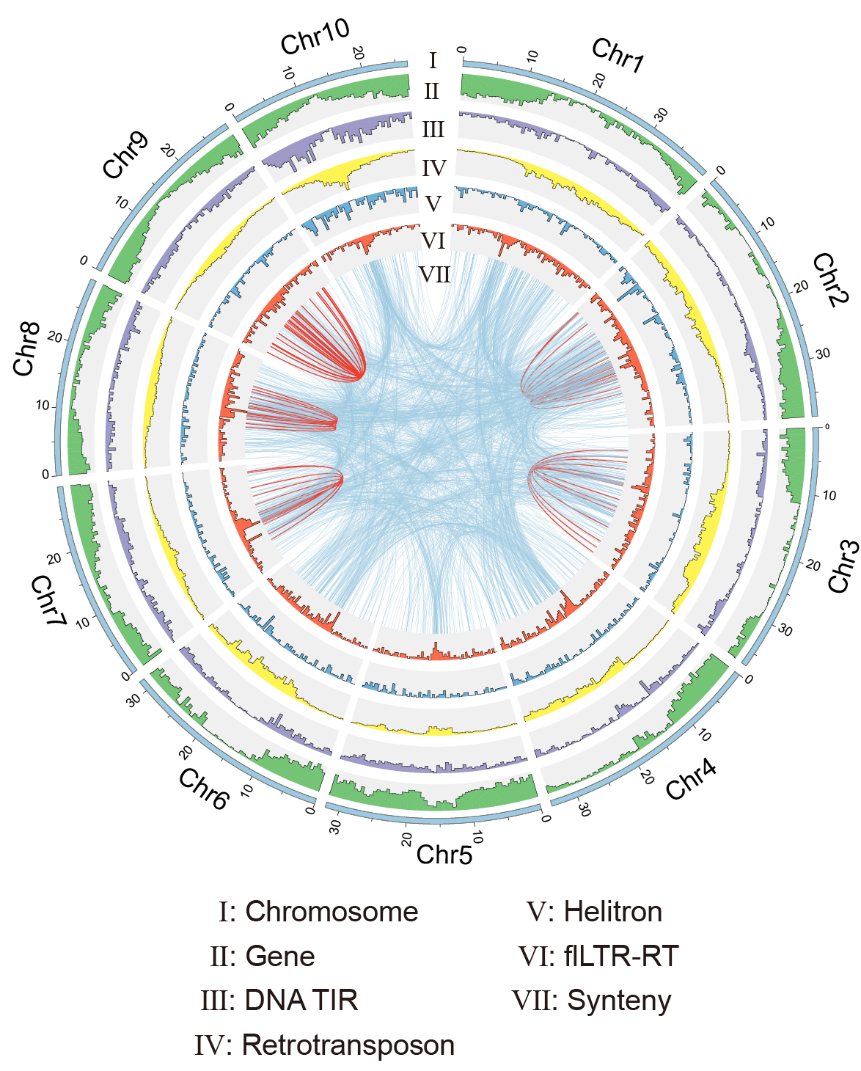


**Figure S3. Landscape of gene and transposable elements in Rc039 genomes.** The outmost circle represents pseudochromosomes and other circles from outer to inner show the genomic density of gene, DNA TIR, retrotransposon, Helitron transposon and LTR-RT. The innermost circle indicates the chromosomal synteny of flLTR-RT within the same chromosome (red line) and between chromosomes (blue line).


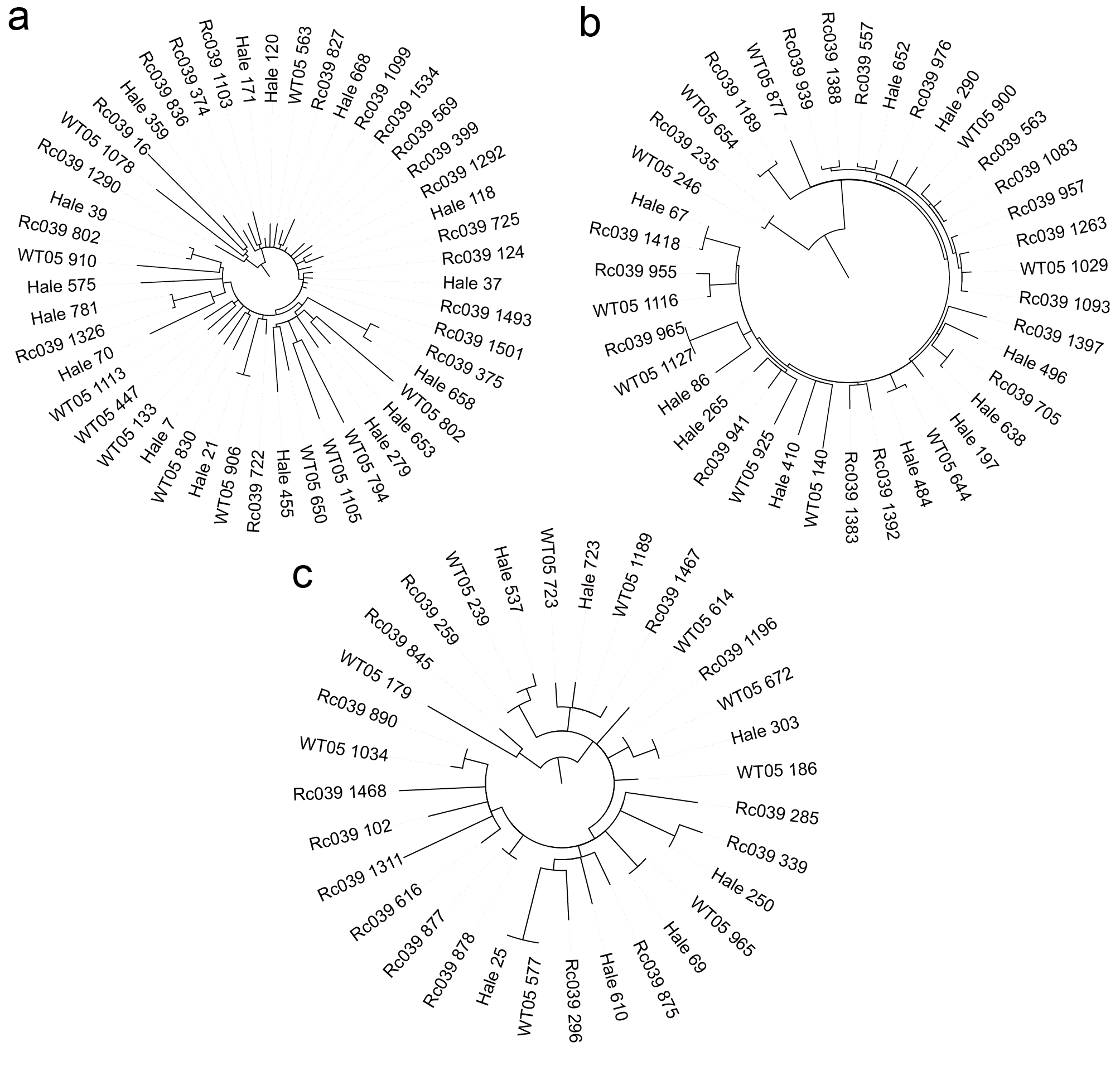


**Figure S4. Phylogenetic tree of the three major full-length LTR-RT families common among Rc039, WT05 and Hale. (a) Top1 cluster (47 copies). (b) Top2 cluster (39 copies). (c) Top3 cluster (32 copies). Node name: accession and flLTR-RT name.**
